# Complete genome of the opportunistic pathogen *Nocardia vulneris* strain LPB4002 and its biosynthetic potential

**DOI:** 10.1128/mra.00840-24

**Published:** 2024-10-29

**Authors:** Janette González-Nava, Leilani I. Salinas-Virgen, Horacio Sandoval-Trujillo, Ma. Eugenia de la Torre-Hernández, Hugo Ramírez-Saad

**Affiliations:** 1 Estancias Posdoctorales por México, CONAHCYT - UAM-Xochimilco, Mexico City, Mexico; 2 Doctorado en Ciencias Agropecuarias, UAM-Xochimilco, Mexico City, Mexico; 3 Departamento de Sistemas Biológicos, Universidad Autónoma Metropolitana-Xochimilco, Mexico City, Mexico; 4 Investigadores por México, CONAHCYT - UAM-Xochimilco, Mexico City, Mexico; Department of Biological Sciences, Wellesley College, Wellesley, Massachusetts, USA

**Keywords:** *Nocardia vulneris*, actinobacteria, genome, biosynthetic genes

## Abstract

The complete genome sequence of the opportunistic pathogen *Nocardia vulneris* is reported. The strain *N. vulneris* LPB4002 was isolated from a clinical sample of a patient with actinomycetoma. The reported genome comprises a single 9,489-Kb closed chromosome, with 8,584 protein-coding genes and 68% GC content.

## ANNOUNCEMENT

*Nocardia* is a genus within the class Actinomycetes, which contains species that are considered pathogenic. Some opportunistic species like *Nocardia vulneris* mainly affect immunosuppressed patients (1–3). Furthermore, Actinomycetes possess a large number of secondary metabolite biosynthetic gene clusters (BGCs), being a source of secondary metabolites with diverse biological activities (4). The most studied BGCs are the polyketide synthase systems (PKSs) and the non-ribosomal peptide synthases (NRPSs) (5).

*Nocardia vulneris* LPB4002 was isolated in 1988 from a patient with actinomycetoma, remaining since then as part of the strain collection at UAM-Xochimilco, Mexico. The strain LPB4002 was identified by 16S rRNA gene sequencing using the primers 27F and 1492R; (27F: 5′-AGAGTTTGATCMTGGCTCAG), (1492R: 5′-TACGGYTACCTTGTTAYGACTT) (6). Strain LPB4002 showed 99.72% similarity and 99% coverage with *N. vulneris* strain NBRC108936^T^ according to the 16S-based ID tool of the EzBioCloud web server (7). The detection of PKS and NRPS systems in the genomic DNA of the strain LPB4002 was done according to Ayuso-Sacido and Genilloud (8). Due to the absence of similar BGC reported in the two available genome sequences of *N. vulneris*, it was decided to sequence the genome of the strain LPB4002.

*N. vulneris* strain LPB4002 was cultured in Sabouraud agar medium (BD Bioxon, Cat. 222400) at 37°C under aerobic conditions for 15 days. The genomic DNA was extracted with the Isolate II Genomic DNA Kit (Bioline, Cat. BIO-52066) following the manufacturer’s protocol. Whole-genome sequencing was performed at Plasmidsaurus (Eugene, OR, USA), using the Oxford Nanopore Technology platform (https://www.plasmidsaurus.com) in a PromethION 24 device. The Library Prep Kit SQK-RBK114.96 kit (ONT) was used, with minimal fragmentation (no ligation, no shearing, and no size DNA selection) of the genomic DNA, which was loaded in a sequence-independent manner in an R10.4.1 flow cell, obtaining >99% data. Base calling was done with ONT Dorado-for-PromethION version 7.1.4 on super-accurate mode. Raw reads with *Q* score < 10 were filtered, and adapters were trimmed by ONT software.

Genome sequencing of *N. vulneris* strain LPB4002 produced 305,972 reads with an average length of 1,980 bp, *Q*30 of 74.9% according to the Phred factor, and 60× coverage. The *de novo* genome assembly was done with Flye version 2.9.1 (9), as implemented in Geneious Prime version 2024.0.4 (10), using the default parameters. These conditions provided a single closed contig of 9,489,173 bp, with 68% GC content. The *N*_50_ value was equivalent to the length of the contig (9,489 Kb) and *L*_50_ = 1. Further genome annotations were done with the PGAP version 6.6 (10). The total number of protein-coding genes was 8,583, with 3 RNA operons containing 50 tRNA genes. The genome of *N. vulneris* has 48 putative biosynthetic genes for PKS and NRPS.

## Data Availability

The genome sequence of *Nocardia vulneris* strain LPB4002 was deposited in GenBank at the following sections (accession numbers): WGS (CP146527); BioProject (PRJNA1080626); BioSample (SAMN40143144), and for the assembly (GCA_037081825.1). The accession number for the 16S rRNA gene sequence is PQ043844.
